# The endocrine disruptor cadmium modulates the androgen–estrogen receptors ratio and induces inflammatory cytokines in luminal (A) cell models of breast cancer

**DOI:** 10.1007/s12020-023-03594-2

**Published:** 2023-11-18

**Authors:** Viviana M. Bimonte, Giuseppina Catanzaro, Agnese Po, Sofia Trocchianesi, Zein Mersini Besharat, Zaira Spinello, Mariaignazia Curreli, Alessandra Fabi, Roberto Bei, Michele Milella, Alessandra Vacca, Elisabetta Ferretti, Silvia Migliaccio

**Affiliations:** 1 Department of Movement, Human and Health Sciences, University of Foro Italico, 00195 Rome, Italy; 2 Department of Experimental Medicine, “Sapienza” University of Rome, 00161 Rome, Italy; 3 Department of Molecular Medicine, “Sapienza” University of Rome, 00161 Rome, Italy; 4 Precision Medicine in Senology Unit, Fondazione Policlinico Universitario A. Gemelli, IRCCS, 00168 Rome, Italy; 5 Department of Clinical Sciences and Translational Medicine, University of Rome “Tor Vergata”, 00133 Rome, Italy; 6 Department of Oncology, University of Verona, 37134 Verona, Italy

**Keywords:** Cadmium, Endocrine disruptors, Breast cancer, Estrogen receptors, Androgen receptor, Cytokines

## Abstract

**Purpose:**

Breast cancer (BC) is the most common malignancy that affects women, and it is, to date, their leading cause of death. Luminal A molecular subtype accounts for 40% of BC and is characterized by hormone receptors positive/human epidermal growth factor 2 expression and current treatment consists of surgery plus aromatase inhibitor therapy. Interestingly, several studies demonstrated that the heavy metal cadmium (Cd), classified as a group 1 human carcinogen and widely spread in the environment, exerts estrogen-like activities in several tissues and suggested an intriguing relationship between increased Cd exposure and BC incidence. Thus, aim of this study was to evaluate effects of Cd on Luminal A BC estrogen receptor (ER) positive/progesterone receptor positive cell models in vitro to characterize the mechanism(s) involved in breast cell homeostasis disruption.

**Methods:**

T47D and MCF7 were exposed to Cd (0.5–1 µM) for 6–24 h to evaluate potential alterations in: cells viability, steroid receptors and intracellular signaling by western blot. Moreover, we evaluated the expression of inflammatory cytokines interleukin by RT-PCR.

**Results:**

Our results showed a significant induction of androgen receptor (AR) and an increased AR/ER ratio. Further, Cd exposure increased pro-inflammatory cytokines interleukin (IL)6, IL8 and tumor necrosis factor α levels. Finally, as previously demonstrated by our group, Cd alters pathways such as mitogen-activated protein kinase family and protein kinase B.

**Conclusion:**

In conclusion, our study demonstrates that Cd modifies the expression and pattern of ERs and AR in BC cell lines, suggesting an alteration of BC cells homeostasis, likely predisposing to a carcinogenetic microenvironment.

## Introduction

Breast cancer (BC) is the most common malignancy that affects women and, despite the improvement in cancer prevention, diagnosis and treatment attained in the last decades, it is, to date, the leading cause of death in women [1]. Luminal A molecular subtype account for 40% of BC and is characterized by hormone receptor positive/human epidermal growth factor 2 expression. Current treatment consists of surgery plus aromatase inhibitor therapy. Among the potential risk factors of BC development and progression, it is well known that long-term exposure to estrogens is linked to increased cell proliferation [2]. The main mediators of estrogenic actions in the target organs are two intracellular receptors, known as estrogen receptor α and β (ERα, ERβ), which belong to the steroid/thyroid intracellular receptors superfamily of transcription factors [3] and act as hormone-dependent transcriptional regulators [4]. Moreover, several studies demonstrated that besides the classical direct nuclear action, an engagement of membrane-ERs might trigger the activation of several rapid extra-nuclear signaling pathways, likely integrating the classic genomic ERs-nuclear-mediated actions [4–7]. Interestingly, experimental evidence indicates that in ERα/β-positive BC, ERα promotes proliferation while ERβ negatively regulates ERα [8]. In addition, recent data suggest that androgen receptor (AR) expression and activity might play a role in BC progression and, indeed, higher AR/ERα ratio is an independent predictor of disease-free survival [9].

Beside long-term estrogenic exposure, several additional potential factors might be responsible for the increased BC incidence in the last decades, even though they are not fully elucidated and characterized. Remarkably, several studies suggest that exposure to environmental pollutants could play a role in the etiology of BC. Indeed, the high incidence of hormone-related cancers and diseases in the Western world has been linked to the presence of pollutants acting as endocrine disruptors (ED) altering physiological hormonal mechanisms and intracellular signaling [10, 11].

Among the different pollutants, the heavy metal cadmium (Cd) is a proven carcinogen [12] and is absorbed into the body from dietary sources, cigarette smoke, and by inhalation in industrial or polluted environments. Cd levels in the human body might increase with age since its removal half-life is quite long (around 10–30 years) [13, 14].

Several data suggest that the main mechanisms of Cd carcinogenesis are initiation of DNA damage, modification of DNA repair mechanisms, promotion of oxidative stress and inflammation, and interference with apoptosis process [15–17]. Furthermore, it has been shown that Cd binds thiols with high affinity and substitutes for zinc in the cysteinyl clusters of many proteins, including ERα and it can also substitute the activity of zinc-depleted transcription factor exerting estrogen-like activity and representing a potent ED. Interestingly, observational studies showed that environmental exposure to Cd is associated with an increased risk of several tumors including BC and that the content of Cd in the tumor tissue of mammary glands is higher as compared to cancer-free, adjacent breast tissue. Stimulation of human BC cell proliferation [15, 16] increased expression of estrogen-regulated genes, activation of ERα [18–20] and increased progesterone receptor levels in BC cells [20] are examples of the estrogen-like effects of Cd.

The aim of our study has been to evaluate the effects of Cd exposure in cellular models of luminal A BC. Cd-induced an increase in the ratio between androgen and estrogen receptors accompanied by an increase in the expression levels of pro-inflammatory cytokines, corroborating the hypothesis of a role of this ED in BC development and progression.

## Materials and methods

### Cell culture and treatment

Breast cancer cell lines, T47D (ATCC HTB-13, Manassas, VA, USA) and MCF7 (ATCC HTB-22), were grown in Dulbecco’s modified eagle’s medium supplemented with 10% Fetal Bovine Serum (FBS), 2 mM Glutamine, 100 units/mL penicillin and 0.1 mg/mL streptomycin. Adherent cells were detached by trypsin-EDTA solution. All cell lines were maintained at 37 °C in a humidified atmosphere of 5 % CO_2_ and 95% air.

In all experimental protocols, T47D cells were seeded at a density of 30,000 cells/cm^2^ and MCF7 cells were seeded at a density of 15,000 cells/cm^2^ in Dulbecco’s modified eagle’s medium without phenol red supplemented with 10% Charcoal stripped (CS) FBS and allowed to grow for 3 days. CS-FBS has low levels of lipophilic compounds, such as hormones, growth factors and steroids, in comparison with FBS. Since Cd is an endocrine disruptor, the hormone deprivation obtained by using this serum allows to better evaluate its effects. Moreover, the absence of phenol red is needed due to the fact that phenol red acts as a weak estrogen stimulating estrogen-sensitive cells [21]. At the end of this treatment, cells were starved 24 h in serum-free medium and treated with Cd 0.5 1, 5, and 10 μM or E2 0.01 µM for different time intervals (6–24 h).

### Reagents

Buffers and reagents for cell cultures were purchased from Corning (Corning, New York, USA) and medium from PAN-Biotech (Aidenbach, DEU). CS-FBS (Cat. #F6765), Cd (Cat. #S-655198-5G) and E2 (Cat. #E2758) were acquired from Sigma Aldrich-MERCK (St. Louis, MO, USA). Cd and E2 were dissolved according to the manufacturer’s protocol and added to cells, as explained in the Cell Culture and Treatment section.

For RNA extraction, the TRIzol RNA isolation reagent was purchased from Ambion™; for reverse transcription, 10 mM dNTP Mix, 50 µM oligodt, RNaseOUT™ Recombinant Ribonuclease Inhibitor, DNase I® and SuperScript® III Reverse were purchased from Invitrogen (Thermo Fisher Scientific, Waltham, MA, USA). SYBR® Green PCR Master Mix for qPCR was purchased from Applied Biosystems™ (Thermo Fisher Scientific).

All reagents for SDS-PAGE were from Santa Cruz Biotechnology (Dallas, TX, USA), Cell Signaling Technology (Danvers, MA, USA), Sigma Aldrich and EuroClone (Milan, Italy)

Primary antibodies were the following: GAPDH (Cat. #MAB374, Sigma Aldrich-MERCK) and AR (Cat. #06-680, Sigma Aldrich-MERCK), ERα (Cat. #sc-542, Santa Cruz Biotechnology), protein kinase B (AKT; Cat. #sc-8312, Santa Cruz Biotechnology), pAKT (Ser473; Cat. #sc-514032, Santa Cruz Biotechnology), extracellular signal regulated kinase (ERK; Cat. #sc-1647, Santa Cruz Biotechnology), pERK1/2 (Thr 44/42, Cat. #sc-7383, Santa Cruz Biotechnology), p38 mitogen-activated protein kinase (MAPK, Cat. #9112, Cell Signaling Technology), pp38 MAPK (Cat. #9211, Cell Signaling Technology), signal transducer and activators of transcription (STAT, Cat. #4904, Cell Signaling Technology) 3 and pSTAT3 (Cat. #9131, Cell Signaling Technology), ERβ (Cat. #ab3576, Abcam, Cambridge, UK). Secondary antibodies were purchased from Jackson Laboratories (Bar Harbor, ME, USA; dilution 1:10.000).

### Trypan blue assay

For trypan blue assay, T47D and MCF7 cells were plated in 30-mm culture dishes and treated with 0.5, 1, 5, and 10 μM Cd or 0.01 µM E_2_ for 24 h. At the end of the incubation with Cd, medium was recovered in a clear tube and adherent cells were detached by trypsin-EDTA solution. After trypsinization, cells were suspended in medium and centrifuged as previously described [22]. Cell viability was determined as the percentage of the total cell number that remained unstained.

### RNA extraction, reverse transcription, and real-time quantitative PCR

RNA extraction was performed using TRIZOL according to the manufacturer’s instructions, as previously described [23, 24]. Treatment with DNAse enzyme was performed to remove genomic DNA contamination. cDNA was obtained by reverse transcription of 500 ng of total RNA. Quantitative real-time PCR was performed in ViiA 7 Real-Time PCR (Thermo Fisher Scientific), using power SYBR green PCR master mix (Thermo Fisher Scientific) as indicated by the manufacturer. Fluorescence intensities were analyzed using the manufacturer’s software (ViiA7 software) and relative amounts of expression gene were evaluated using the 2−^∆∆Ct^ method and normalized for –β-actin. Data are expressed as fold increase. The primer sequences are summarized in Table 1 and gene expression of TNF-α and β-actin was assessed using Applied Biosystems™ “best coverage” assays (Thermo Fisher Scientific).

**Table 1:** Human-specific primers pair sequence for RT-PCR analysis

Gene	Forward	Reverse
β-actin	CCCAGATCATGTTTGAGACCT	GAGTCCATCACGATGCCAGT
Interleukin (IL) 8	TCC TGA TTT CTG CAG CTC TGTG	GTC CAG ACA GAG CTC TCT TCCAT
Interleukin (IL) 6	TTC GGT ACA TCC TCG ACG GC	TCT GCC AGT GCC TCT TTG CT

### Protein extraction and western blot analysis

After each treatment, cells were washed with phosphate-buffered saline and lysed in fresh ice-cold RIPA buffer (150 mM NaCl, 50 mM Tris–HCl, pH 7.5, 500 µM EDTA, 100 µM EGTA, 1.0% Triton X-100 and 1% sodium deoxycholate) supplemented with a cocktail of protease and phosphatase inhibitors (Sigma Aldrich). For immunoblot analysis, an equal amount of proteins (30 µg) was resolved in SDS-polyacrylamide gels (8–12 %) and transferred onto nitrocellulose membranes (GE Healthcare, Chicago, IL, USA) as previously described [25]. Thereafter, membranes were incubated with primary antibodies appropriately diluted. All primary antibodies were diluted in 1× Tween Tris-buffered saline with 5% bovine serum albumin or 5% non-fat dry milk according to the manufacturer’s protocol and incubated over night at 4 °C. All Santa Cruz Biotechnology primary antibodies were diluted 0.2 µg/ml, Cell Signaling Technology and MERCK antibodies were diluted 1:1000. Proteins were revealed by enhanced chemiluminescence (LiteAblot® TURBO; EuroClone). Densitometric analysis of the bands was performed using Image J Software v1.51 (NIH, Bethesda, MD, USA) using GAPDH for normalization.

### Statistical analysis

Statistical analysis was performed by using a multiple *t* test or One- way Anova followed by a Bonferroni-Dunn post-hoc test (GraphPad Prism, San Diego, CA, USA). Data are presented as mean ± SEM or as fold increase vs. untreated cells of at least three independent experiments. *P* values < 0.05 were considered statistically significant.

## Results

### Cadmium exposure modulates expression pattern of steroid receptors in Luminal A cell models

Since our previous results [18] demonstrated that Cd can alter BC cell homeostasis in vitro by an ER-mediated mechanism, experiments were performed to evaluate whether this pollutant could modify the expression pattern of ERα, ERβ, and AR in ER + BC cells. To this end, we performed experiments in the Luminal A cellular models: T47D cells and MCF7, both positive for ERα and progesterone receptors.

**Fig. 1:**
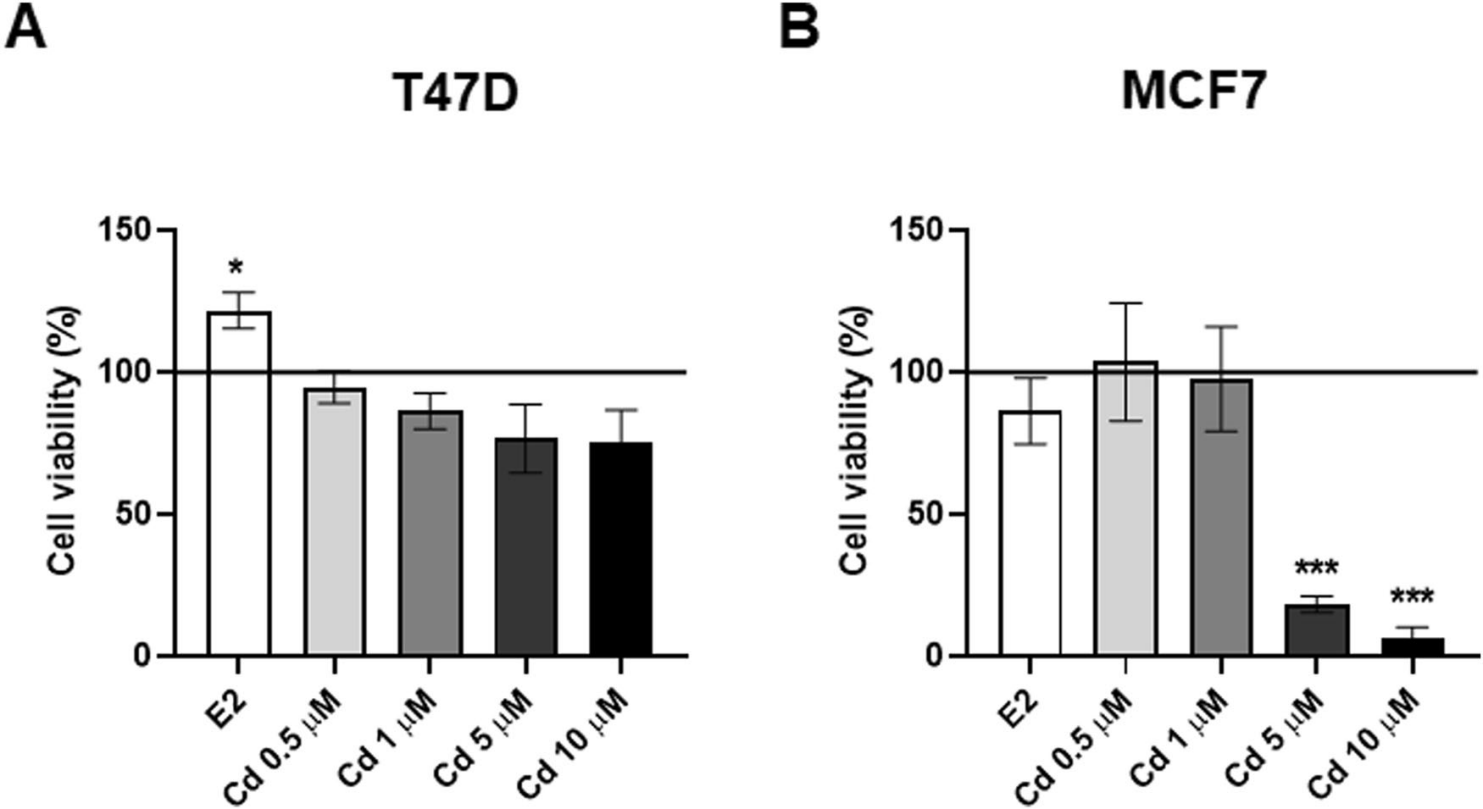
Cadmium (Cd) effects on cell viability. Evaluation of T47D (**A**) and MCF7 (**B**) cell viability by trypan blue assay, upon exposure to increasing Cd concentrations (0.5, 1, 5, and 10 µM) for 24 h (hrs). Results are represented as mean ± standard error mean (SEM) of three independent experiments (*n* = 3). The percentage of cell viability is reported relative to untreated cells, defined as 100 and indicated with a black line parallel to the *x*-axis. E2, Estradiol. *p < 0.05 and ***p < 0.001 *vs* CTRL

First of all, to assess optimal non-cytotoxic concentrations of Cd to be used, BC cells were exposed to Cd and cell viability was assessed by trypan blue assay (Fig. 1). Both T47D and MCF7 cells were incubated in the presence or absence of 0.5–10 μM Cd for 24 h. Since 5 and 10 µΜ Cd concentrations significantly affected BC cells viability, especially in MCF7 cells (Fig. 1), 0.5 and 1 μM Cd concentrations were chosen and used for all the subsequent experiments.

**Fig. 2:**
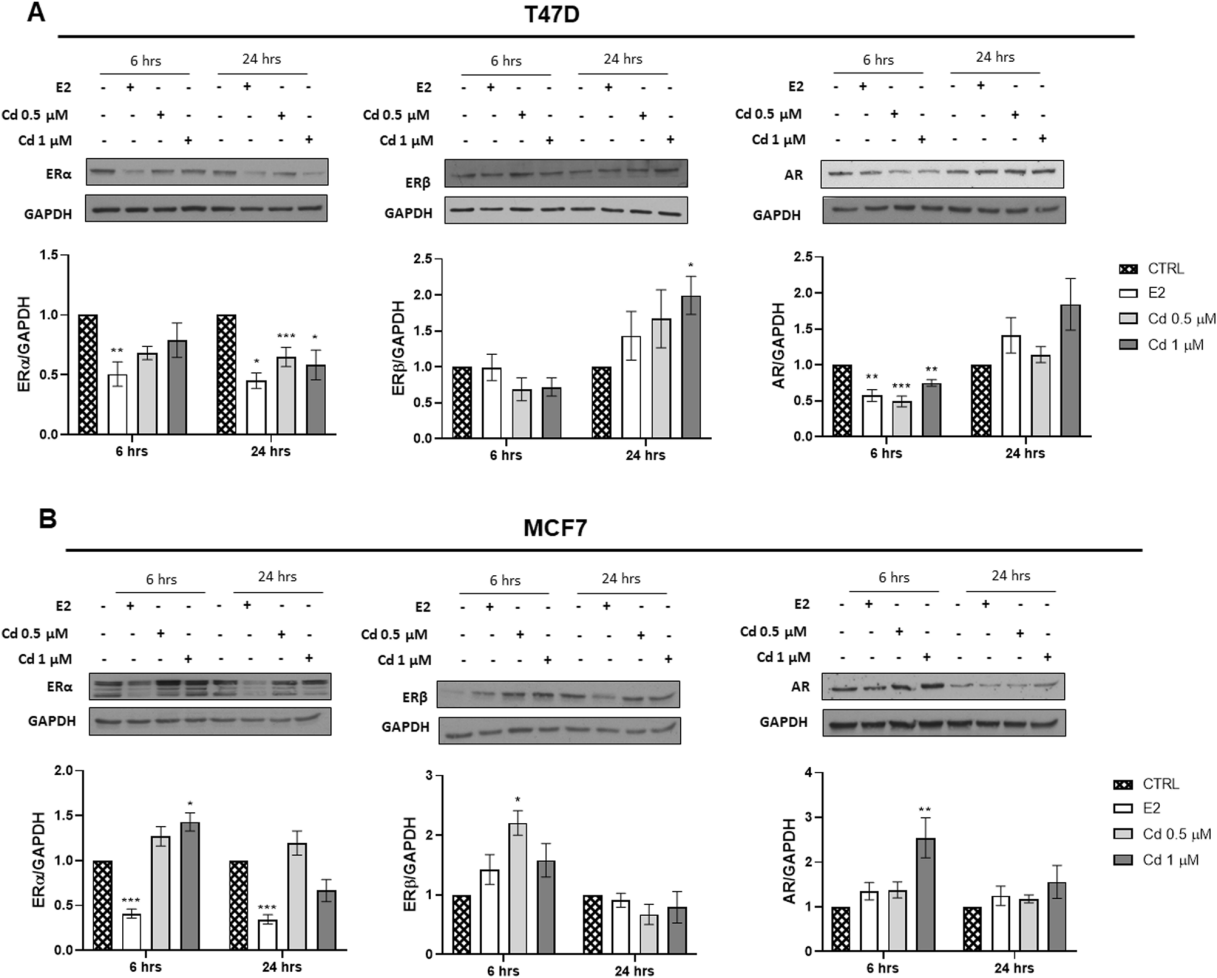
Cadmium (Cd) effects on protein expression level of sex steroid hormones receptors. Figure 2 depicts a representative western blot of the effects of Cd (0.5–1 μM) on ERα, ERβ and AR protein levels in T47D (**A**) and MCF7 (**B**) cell lines. Each cell line was treated in the absence or presence of Cd (0.5–1 µM) or E2 (0.01 µM) for 6–24 h (hrs). Protein loading control: glyceraldehyde-3-phospahte dehydrogenase (GAPDH). Results are represented as mean ± SEM of three independent experiments. *p < 0.05, **p < 0.01 and ***p < 0.001 *vs* CTRL

We then proceeded to investigate the effects of Cd on the expression of steroid receptors. To this aim, BC cells were incubated in the presence or absence of 0.5–1 μM Cd or estradiol (E2) 0.01 μM for 6 and 24 h. Interestingly, Cd effects on ERα and β were partially different from the effects induced by E2. Exposure of T47D to Cd 0.5 and 1 µΜ significantly decreased ERα levels after 24 h in a manner similar to E2, while inducing an increase of ERβ expression (Fig. 2A). Interestingly, AR protein expression level was decreased after 6 h exposure, while not significantly affected after 24 h (Fig. 2A). Conversely, MCF7 cells showed an increase of all the investigated steroid receptors (ERα, ERβ, and AR) after 6 h exposure (Fig. 2B). As expected E2 significantly decreased ERα, as previously demonstrated [18]. Our data indicate that in T47D cells, Cd affects first AR and subsequently ERs. Conversely, in MCF7 cells the three receptors are synchronously induced. These data suggest that the two luminal A BC cells react differently to Cd.

### Cadmium exposure modulates expression pattern of steroid receptors in Luminal A cell models

**Fig. 3:**
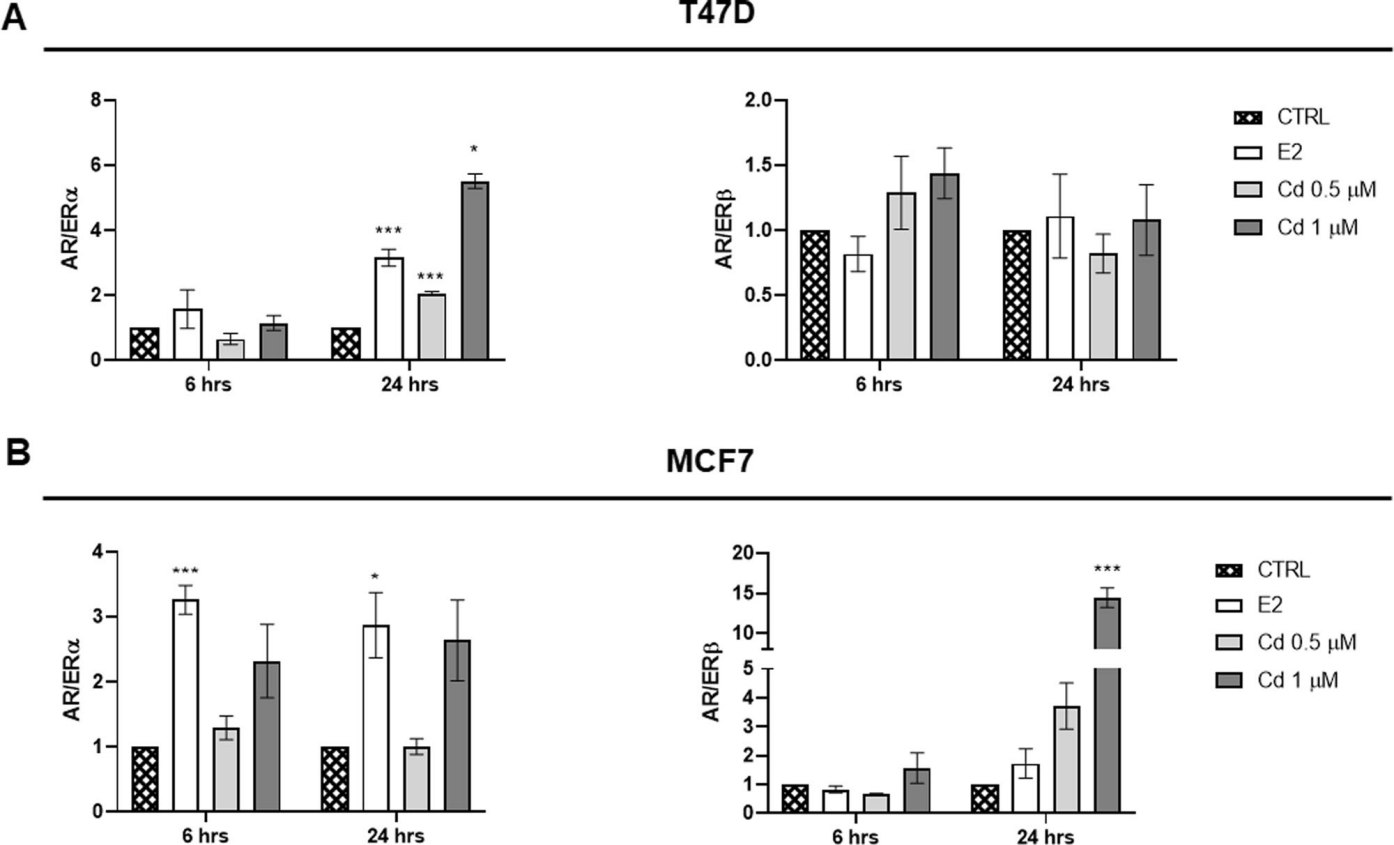
Cd effects on AR/ERα and AR/ERβ ratio. Results are expressed as AR/ERα and AR/ERβ ratios in T47D (**A**) and MCF7 (**B**) cells lines as mean of three independent experiments performed in triplicate *p < 0.05, ***p < 0.001 vs CTRL

Since patients with AR/ER ratio ≥2 show worse disease-free survival in the presence of anti-estrogen therapies or chemotherapy treatment compared to patients with lower ratio [26, 27], we wondered if Cd could alter the AR/ER ratio in our BC cellular in vitro models. Interestingly, Cd-induced a significant increase in AR/ERα in T47D, without any effect on AR/ERβ ratio (Fig. 3A). Conversely, Cd did not affect the AR/ERα, while significantly increased AR/ERβ ratio in MCF7 (Fig. 3B), likely suggesting a different responsiveness of BC cells to hormonal stimuli and supporting the hypothesis that the two BC models are differently affected by Cd.

### Cadmium exposure affects PI3K/AKT, ERK1/2, and p38 MAPK signaling pathways in Luminal A cell models

**Fig. 4:**
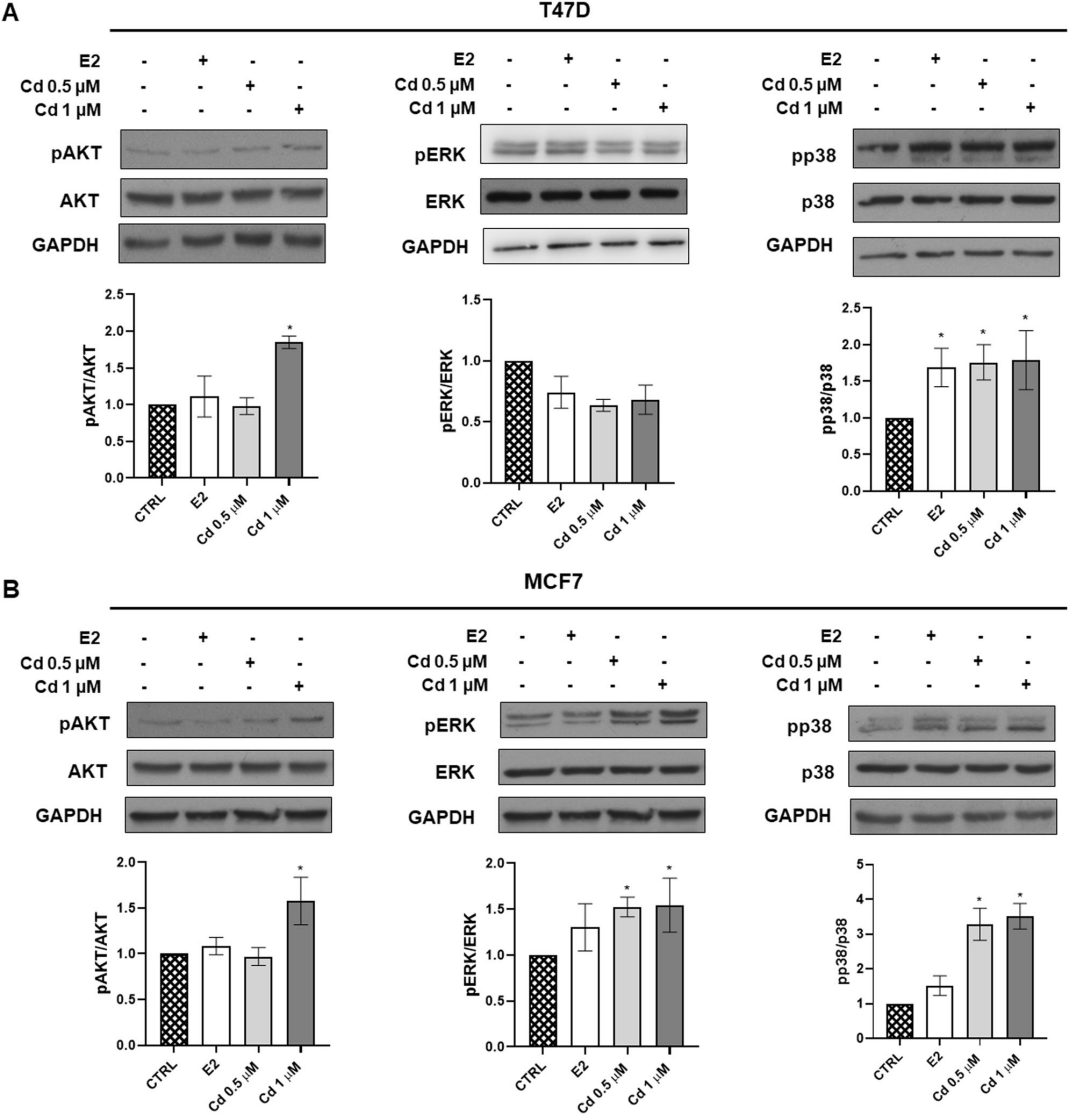
Cd effects on PI3K and MAPK signaling pathways. The figure depicts representative western blots of the effects of Cd (0.5–1 μM) on phospho-AKT (pAKT), phospho-ERK (pERK), phospho-p38 (pp38) in T47D (**A**) and MCF7 (**B**) cells after 6 h of exposure. Phosphorylated proteins were normalized to each respective level of total protein. Protein loading control: GAPDH. Results are shown as mean ± SEM of three independent experiments. *p < 0.0 5 vs CTRL

To further characterize the biological effects of Cd, the two different Luminal A cell lines were exposed to 0.5 and 1 μM Cd for 6 h and the effect of this exposure on the phosphoinositide 3-kinase (PI3K)/AKT and MAPK pathways were evaluated, since alteration of these pathways is often present in this type of tumor. As shown in Fig. 4A, AKT and p38 phosphorylation increased after treatment with Cd in T47D, while ERK1/2 phosphorylation was not affected. In MCF7 instead Cd exposure significantly activated all these pathways (Fig. 4B), confirming our previously published results [18]. In addition, these data underline that Cd activates PI3K/AKT, ERK1/2, and p38 MAPK signaling pathways and further corroborate the different sensitivity of the two luminal A BC models to this ED.

### Cadmium stimulates the transcription of the pro-inflammatory cytokines IL-6, IL-8, and TNFα and activation of STAT signaling pathway

**Fig. 5:**
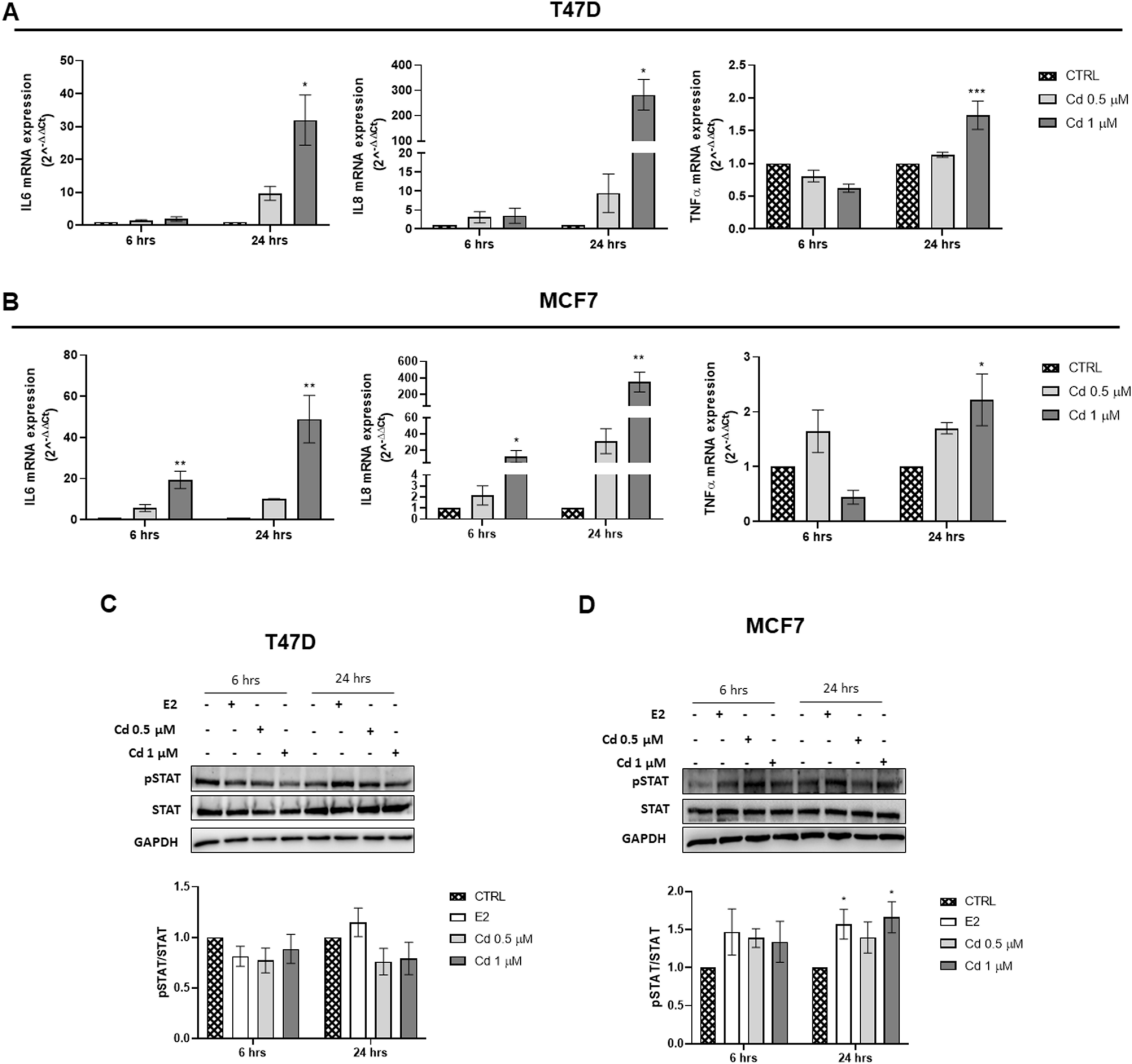
Cd effects on inflammation markers and STAT signaling. Evaluation of mRNA expression levels of IL6, IL8, and TNFα and of STAT3 phosphorylation (pSTAT) in T47D (**A**, **C**) and MCF7 (**B**, **D**) cells incubated with or without Cd (0.5 and 1 µM) for 6–24 h. Gene expression levels were normalized to β-actin. pSTAT was normalized to the respective level of the total protein. Protein loading control: GAPDH. Results are shown as mean ± SEM of three independent experiments performed in triplicate. *p < 0.05, **p < 0.01 vs CTRL

Since Cd has been described to increase inflammatory markers in different cellular contexts [28, 29], and it is known that inflammation influences the development of cancer and promotes all stages of tumorigenesis [30], we evaluated whether Cd could increase pro-inflammatory cytokines levels, such as IL6, IL8, and TNFα in both Luminal A BC cell lines. To this end, T47D and MCF7 cells were incubated in the presence or absence of Cd 0.5–1 μM for 6 and 24 h. As shown in Fig. 5A, in T47D cells, Cd 1 μM induced a significant increase of IL6, IL8, and TNFα after 24 h. In MCF7 cells the significant increase of both IL6 and IL8 was evident already after 6 h exposure, while TNFα increase was significant after 24 h (Fig. 5B). Since one of the most relevant transcription factors families in BC is the STAT family [31], we wondered if Cd exposure induced an increase in STAT3 phosphorylation (pSTAT3). As reported in Fig. 5, pSTAT3 activation in T47D was not affected at the time points analyzed, while it was increased in MCF7 after 24 h of Cd exposure. These data suggest that Cd induces a marked pro-inflammatory state that can promote BC development and progression.

## Discussion

Environmental pollution by Cd is a worldwide severe health issue, particularly due to its long half-life estimated to be 10–30 years [32] and, additionally, for the lack of an effective therapy for Cd harm. In vitro experiments performed in BC cells indicate that at low concentrations, Cd stimulates DNA synthesis and cell proliferation [17] but, depending on both the exposure conditions and the experimental model used, it might also induce alteration in cell homeostasis [17]. Although Cd has been demonstrated to induce toxicity, the precise mechanism(s) remain poorly understood. Nevertheless, knowledge of the mechanisms by which Cd exerts its toxic effect on specific tissues is mandatory not only to understand how this ED might induce homeostasis cells disruption, but also to develop new therapeutical strategies.

In particular, observational clinical studies showed that environmental exposure to Cd is linked to an increased risk of BC [33, 34], nevertheless several gaps are still present in the knowledge of the mechanisms involved in Cd-related increase of BC [17].

Thus, the present study was performed to further characterize the mechanism(s) underlying the potential alterations induced by Cd in Luminal A BC cells in vitro. Specifically, we used two different cellular models of Luminal A BC, namely T47D and MCF7 cells. According to literature data, they differently express the three sex-hormone receptors (ERα, ERβ, and AR). In both cell lines, ERα is the most abundant expressed receptor, followed by AR and finally ERβ [35]. In particular, AR was much more expressed than ERα in T47D cells in comparison with MCF7, suggesting the AR may have a most important role in T47D than in MCF7 cells [35]. Additionally, these two cell lines show other different features (i.e., T47D are p53 mutant, whereas MCF7 are p53 wild type) that could explain the different responses we observed after Cd exposure [35, 36].The results of the present research further corroborate our previous data showing that Cd can modulate ERα expression not only in MCF7 cells, but also in another Luminal A BC cellular model system as T47D, suggesting that ERα alteration upon the exposure to this pollutant is a widespread event in BC cells. Also ERβ expression was significantly modulated in the two BC cell lines. In both cases, the two ERs were modulated with a different timing, being T47D less responsive at earlier time points than MCF7 cells. Interestingly, ERβ has been closely linked to BC progression but classic hypotheses regarding ERβ as a tumor suppressor, has indeed been confuted by more recent studies [37]. Of note, recent results by Suzuki demonstrated that 4-methyl-2,4-bis(4-hydroxyphenyl)pent-1-ene (MBP), an active metabolite of bisphenol A, an environmental pollutant with estrogenic activity, significantly decreases ERα expression in ERα/β-positive human BC, yet stimulating cell proliferation through the activation of ERβ-mediated transcription [8], supporting the concept that exposure to EDs might alter the functional role of ERβ in BC cells from a suppressor to a promoter role. Our in vitro results further suggest that Cd, as other EDs might alter cancer responsiveness by an alteration of ERα and ERβ in the cells, but deeper studies are needed to dissect this specific role of ERβ as either suppressor or promoter and clarify the role of ERβ in BC progression.

More interestingly, our data indicate, for the first time, that Cd exposure of Luminal A BC cells increases the AR/ER ratio. The AR is a steroid hormone receptor widely detected in BC, but its role in BC tumorigenesis, proliferation, and progression is still not fully characterized. The results presented herein appear quite interesting and intriguing since data obtained in women affected by BC suggest that an increased level of AR might lead to a more aggressive phenotype. Clinical studies indeed indicate that BC distant recurrence appears significantly higher in AR-positive patients (91.3% versus 33.3% of all recurrences) [38]. The role of AR in metastasis development includes coordinated actin filament dynamics mediated by cofilin and other connected proteins. Additional in vitro data showed that androgens enhanced cell polarization, stimulated wound healing and transwell migration rates and increased N/E-cadherin mRNA ratios [39]. Other evidence suggests that the AR might be a tumor suppressor in ERα -positive (BC, but a tumor promoter in ERα negative BC [40]. The increased AR/ER ratio in BC cells induced by Cd exposure might then suggest a role of this pollutant in inducing a more aggressive phenotype by alteration of androgen and estrogen receptor pattern since activated AR might contribute to induce a more aggressive phenotype of BC cells.

Moreover, our data confirm our previously published results demonstrating that this heavy metal activates AKT, leading to a disruption of homeostatic signaling of BC cells [41]. Remarkably, alteration of AKT activity might increase chemotherapeutic drug and hormonal resistance in BC cells [41] and these novel data suggest that Cd might further influence hormonal resistance leading to a more aggressive phenotype of BC by altering this specific signaling pathway. Additionally, both ERK1/2 and p38 MAPK activation may favor an aggressive BC phenotype. It has been reported indeed that they are both able to modulate specific molecules involved in the metastatic process by regulating crucial players of the homeostatic control of the extracellular matrix [42]. Furthermore, p38 MAPK favors BC growth and metastatization by acting on the tumor microenvironment and enhancing tumor vascularization through the increase of several pro-angiogenic cytokines, such as vascular endothelial growth factor A and IL6 [43]. Finally, ERK1/2 is hyper-expressed [44] and hyper-activated in BC in comparison with normal tissue [45].

Another interesting result is the significant increase of the pro-inflammatory cytokines that are synthesized in physiological and pathological conditions, and secreted by different cell types (immune cells, immunocompetent cells, some cancer cells) [46]. It is known that inflammation is an important detrimental factor in cancer and inflammatory BC, related to high level of cytokines, represents a deadly aggressive phenotype of BC with a specific clinicopathological presentation and low survival rate. Several cytokines, including leptin, IL-1B, IL-6, IL-8, IL-23, IL-17, and IL-10, stimulate cancer progression, while others, including IL-2, IL-12, and IFN-γ, inhibit cancer proliferation and/or invasion [46]. The pleiotropic effects of cytokines in tumorigenesis can explain the cross-talk among the pathways that regulate tumor progression, such as Janus kinase (JAK)/STAT, PI3K, AKT, Rac, MAPK, nuclear factor kappa-light-chain-enhancer of activated B cells (NF-κB), cFos and mammalian target of rapamycin (mTOR) [46]. As an example, TNFα is involved in tumor promotion, aggressiveness, infiltration of tumor promoting macrophages, angiogenesis and IL-8 expression. Additionally, invasion is directly proportional to IL-8 expression in several breast cancer cell lines [46, 47] and its expression in breast cancer appears to be induced by other cytokines (IL-1β, TNFα and IL-6), an/or hormones (progesterone and estrogen) [46–48]. Specifically, our data showed an increase of IL6, IL8, and TNFα upon exposure of both cell type models to Cd.

Of note, IL6 and IL8 are two of the cytokines involved in these more aggressive BC phenotypes [49]. In particular, IL6 induces proliferation and a more aggressive phenotype in ER+ cells [50]. Additionally, IL6 promotes the conversion of non-stem cancer cells into cancer stem-like cells, an inducible model of breast oncogenesis [51]. Sullivan and others also showed as the overexpression of IL6 in MCF7 cells induced the epithelial-mesenchymal transition and increased their invasiveness [52, 53]. In BC, IL8 is associated with metastatization, endothelial cells proliferation as well as lymph node positivity and tumor aggressiveness [54]. Furthermore, serum IL8 has been linked with fast clinical progression, greater tumor bulk and more advanced disease [55]. Finally, TNFα is highly expressed in BC and induces the proliferation of T47D cells by an increase of cyclin D, through an activation of NF-kB pathway [54]. Several cytokines may be responsible of STAT activation and STAT3 has been closely associated with cancer aggressiveness [30] in BC. Interestingly, STAT3 activation by IL6/JAK 2 pathway may inhibit apoptotic pathways activation [56] as well as favors metastatization through the up-regulation of matrix metalloproteinases as well as mesenchymal markers [57, 58]. Of note, we observed an increase in the active form of STAT3 only in MCF7, therefore we hypothesize that other signaling pathways are activated downstream in T47D cells and envisage the need of further research to better define the molecular mechanisms associated to Cd effects in different Luminal A BC models. Last, Cd, as other EDs, has been defined as pro-inflammatory inducer in several different tissues, such as human endothelium, kidney, testis [29, 59, 60]. Thus, the increase of these cytokines and the activation of the transcription factor STAT3 suggest that this environmental pollutant might also alters the inflammatory pattern in the microenvironment of mammary tissue, leading to a more aggressive BC phenotype.

## Conclusions

In conclusion, our results suggest that Cd, as other pollutants with estrogenic activity, can disrupt breast cell homeostasis triggering signals otherwise switched off. In particular, the activation of the signaling linked to cell proliferation, as well as the inflammatory status and the alteration of ERs and AR pattern induced by Cd could in part play a role in BC progression and aggressiveness. However, further studies are required to fully clarify the role of this pollutant in BC carcinogenesis in order to identify novel potential targets for pharmacological therapeutic intervention.
